# Obligatory medical prescription of antibiotics in Russia: Navigating formal and informal health‐care infrastructures

**DOI:** 10.1111/1467-9566.13224

**Published:** 2021-02-26

**Authors:** Alena Kamenshchikova, Marina M. Fedotova, Olga S. Fedorova, Sergey V. Fedosenko, Petra F. G. Wolffs, Christian J. P. A. Hoebe, Klasien Horstman

**Affiliations:** ^1^ Department of Health, Ethics and Society School of Public Health and Primary Care (CAPHRI) Maastricht University Maastricht The Netherlands; ^2^ Department of Faculty Paediatrics Siberian State Medical University Tomsk Russian Federation; ^3^ Department of General Medical Practice and Outpatient Therapy Siberian State Medical University Tomsk Russian Federation; ^4^ Department of Medical Microbiology School of Public Health and Primary Care (CAPHRI) Maastricht University Medical Center (MUMC+) Maastricht The Netherlands; ^5^ Department of Social Medicine and Medical Microbiology School of Public Health and Primary Care (CAPHRI) Maastricht University Maastricht The Netherlands; ^6^ Department of Sexual Health Infectious Diseases and Environmental Health South Limburg Public Health Service (GGD South Limburg Heerlen The Netherlands

**Keywords:** antimicrobial resistance, antibiotics, Russia, health policy, behaviour, infrastructure

## Abstract

Antimicrobial resistance control programmes often aim to “fix” the behaviour of antibiotic users and prescribers. Such behavioural interventions have been widely criticised in social science literature for being inefficient and too narrow in scope. Drawing on these criticisms, this article analyses how political programmes for fixing antibiotic behaviours were adapted into the practices of health‐care professionals and patients in Russia. In 2018, we conducted interviews with medical doctors, pharmacists and patients in a Russian city; focusing on their practices around the policy requirement introduced in 2017 which obligated medical prescriptions of antibiotics. We conceptualised the obligatory medical prescription as a political technique which sought to change practices of self‐treatment and over‐the‐counter sales of medications by establishing doctors as an obligatory passage point to access antibiotics. Our analysis shows that the requirement for medical prescriptions does not fulfil the infrastructural gaps that influence antibiotic practices. By navigating the antibiotic prescriptions, doctors, pharmacists and patients informally compensate for the gaps in the existing infrastructure creating informal networks of antibiotic care parallel to the requirement of obligatory prescriptions. Following these informal practices, we could map the inconsistencies in the current policy approaches to tackle AMR as a behavioural rather than infrastructural problem.

## INTRODUCTION

Control and regulations of antibiotics in health care have been a priority for national and international programmes for addressing antimicrobial resistance (AMR) including prescriptions, over‐the‐counter sales and self‐medication (Chandler 2019, Will 2018). In the 2015 Global Action Plan, individual behaviour regarding antibiotic use is identified as one of the leading causes of AMR (World Health Organization 2015). Following the international lead, national policies against AMR have focused on reducing “unnecessary use” of antibiotics in health care (European Commission 2017, O'Neill 2016). However, the predominantly behavioural focus of AMR policies has been scrutinised by various social scientists who highlight their narrow capacity and limited effectiveness (Haenssgen *et al*. 2018, Pearson and Chandler 2019). Scholars like Chandler (2019) and Will (2018) argue that it is necessary to understand the underlying social, economic and political processes that influence antibiotic practices, rather than focusing on the individual behaviour of users.

Despite this criticism, behavioural interventions have continued to play an essential role in AMR control programmes (Broom, Kenny, Prainsack, *et al*. 2020). To understand the work and the role of these interventions within the established health‐care infrastructures, we studied practices of medical doctors, pharmacists and patients in a Russian city. The Russian context is particularly interesting because AMR is a relatively new object in the policy agenda of this country, and over‐the‐counter sales of antibiotics were not prohibited until 2017 (Government of the Russian Federation 2017). Our research was conducted amidst policy changes that were aiming to influence antibiotic practices. Following scholars like Chandler (2019) and Landecker (2015), we conceptualised antibiotic practices both as an integral part and a result of health‐care infrastructure. In this work, the concept of infrastructure is based on the tradition of science and technology studies (Bowker and Star 2000). The infrastructure is understood as both the material infrastructure (e.g. laboratory equipment) and the relational infrastructure, which refers to the coordination and communication between different health actors.

To analyse this health‐care infrastructure in a context of recent policy changes, we focused on how practices of medical doctors, pharmacists and patients have adapted to the implementation of obligatory standardised antibiotic prescription as part of the national action plan against AMR introduced in 2017. Contributing to discussions on behavioural interventions against AMR, we analysed the obligatory standardised medical prescription in Russian health care as a political technique to manage antibiotic behaviour within the established health‐care infrastructure.

After introducing the theoretical background of our study, we describe our research setting and methodology. In the results following, we present the analysis of how obligatory medical prescriptions mediate the practices of doctors, pharmacists and patients in a Russian city. Our discussion section reflects upon the findings in the context of current theoretical debates to control individual antibiotic behaviour.

## THEORETICAL BACKGROUND

Behavioural interventions including awareness campaigns for health‐care professionals and patients, practices of antibiotic surveillance, and control over individual behaviour have been deliberately scrutinised by social science scholars. The research conducted by Pearson and Chandler (2019) in Ethiopia, India, Nigeria, the Philippines, Sierra Leone and Vietnam demonstrates that awareness regarding AMR among health‐care professionals does not automatically translate towards reduction of antibiotic prescriptions. The authors show that despite AMR awareness, health‐care professionals prescribe antibiotics due to infrastructural constraints, such as lack of diagnostics and shortages of medical staff; social constraints, such as economic access to antibiotics; and overall level of hygiene and sanitation in communities. Pearson and Chandler (2019) argue that rather than reducing antibiotic prescriptions, high level of AMR awareness among health‐care professionals has given them confidence in prescribing broad‐spectrum antibiotics, believed to be more effective in settings with poor hygiene and limited diagnostic capacity.

In a special issue of this journal, Will (2018) scrutinises individual behaviour interventions as a solution for AMR and emphasises the need for more nuanced understanding of the “routines and logic” that shape organisations and delivery of care. She elaborates that antibiotic prescription behaviour of medical doctors can be influenced by multiple processes, including their relationships with particular patients, the socioeconomic status of these patients, their communication with colleagues, and their feelings of responsibility towards an individual patient and society as a whole. Following criticism of the behavioural approach to AMR, Chandler (2019) argues that the focus on behavioural changes tends to overlook infrastructural conditions that can influence the behaviour of antibiotic users. She elaborates that the question of antibiotic use is not a simple rational question of choice and availability of knowledge, often argued by behaviour‐oriented policies; but it depends on larger infrastructural processes that define social, economic and political inequities.

Case studies from different parts of the world demonstrate how antibiotic practices are the results of adapting relations that health‐care actors build around the organisation of material infrastructures. Studying the social world of urban Australian hospitals, Broom *et al*. (2014) show that AMR does not necessarily play a significant role in clinical decision‐making. Rather, doctors make decisions on antibiotic prescriptions following the “rules of the game” within a hospital. These rules can reflect professional hierarchies between more and less experienced doctors, and a constant negotiation of immediate clinical risks versus long‐term population burdens. In a similar study in a rural Australian hospital, Broom *et al*. (2017) show that doctors adapt their prescription practices to the social and infrastructural realities of the rural area. Such realities may include mobility of patients (e.g. those who come to rural areas for seasonal work), accessibility of health‐care services (e.g. large distances between a hospital and residential areas) and conditions that challenge the continuity of treatment and thus may stimulate prescriptions of antibiotics. Several studies state that antibiotic prescriptions can be influenced by intricate relationships between doctor and patient, as well as pharmacist and patient (Broom, Kenny, Kirby, *et al*. 2020, Cabral *et al*. 2015, Lambert *et al*. 2019). Depending on how health‐care professionals perceive the expectations of patients, they may feel obliged to prescribe or sell antibiotics to preserve a trusting doctor–patient relationship and provide a physical symbol of care in the form of a prescription.

Antibiotic practices of patients can also be influenced and shaped by different processes beyond immediate knowledge on AMR. Research by Willis and Chandler (2019) with participants from Tanzania and Uganda demonstrated that behavioural practices are influenced by structural dimensions. Such dimensions include limited access to non‐pharmaceutical forms of care, economic demands for productivity, and thus inability to sufficiently convalesce after an illness and poor sanitary conditions in certain settings. These of which can influence people to turn to antibiotics as a form of prevention. Willis and Chandler (2019) argue that within these dimensions antibiotic use can be understood as an attempt to quickly “fix” access to care, individual productivity and poor hygiene.

These diverse examples highlight the essential role of critical analysis for understanding policy interventions aiming to “fix” the behaviour of antibiotic users. Antibiotic practices are embedded and shaped by the material and relational infrastructures of health care, policy and economic systems (Chandler 2019, Landecker 2015). Drawing from this, Chandler (2019) highlights how antibiotics are embedded into the different spheres of modern living: defining hygiene as non‐bacterial, health care as based on pharmaceutical care, and productivity as not disrupted by prolonged illnesses. AMR and policies aiming to tackle it, she argues, are performing disruptions of these infrastructures by challenging conventional practices of antibiotic use and making visible the dependencies between these medicines and modern ideas of health and productivity.

Building on the idea that antibiotic practices are embedded and determined by the existing health‐care infrastructure, we conceptualise obligatory medical prescription in Russia as a potential disruption or inversion of this infrastructure that allows us to understand the current practices of care delivery. The obligatory medical prescription is understood here through the concept of boundary object. This concept was originally developed by Star and Griesemer (1989) in their analysis of the Museum of Vertebrate Zoology at the University of California. The authors argue that the notion of boundary objects facilitates understanding of how the complex work of diverse actors can be performed. An example of a boundary object is, for instance, a standardised system for disease classification (Bowker and Star 2000). Such a system is simple enough that different actors including medical doctors, technicians, nurses and insurance agents can navigate it. Yet, at the same time, the coding can provide specific information relevant to each of the actors: such as the line of treatment for medical doctors, and information on applicability to particular insurance policies for the insurance agents. As such, this system aligns the work of both. Focusing on the practices of medical doctors, pharmacists and patients, here we will disentangle the current antibiotic infrastructure and make visible the practices and dependencies that antibiotics create in Russian health care.

## AMR, ANTIBIOTICS AND RUSSIAN HEALTH CARE

Following the global movements in developing national plans to tackle AMR, Russia launched its national strategy to prevent the spread of AMR from 2017 to 2030. Similar to the Global Action Plan on AMR, the Russian strategy highlights the importance of tackling the misuse of antibiotics in health‐care practices, articulating the need for behavioural changes among antibiotic users and prescribers. Despite prohibition on the sale of antibiotics without a prescription since 2006, no mechanisms existed to enforce this. Since 2017, the federal government introduced several changes into the federal regulation on drug circulation (Federal law №61). In particular, surprise inspections of pharmacies (i.e. unplanned and unnotified) have been implemented to enforce control of over‐the‐counter sale of medications, including antibiotics (Federal law №61, Article 9). In addition, pharmacists have been obliged to collect and file patients' prescriptions for inspection purposes (Decree N 647н). The introduction of surprise inspections and the requirement to collect and file patients' prescriptions in a pharmacy in 2017 restricted the over‐the‐counter sale of medications and thus necessitated patients to seek an official standardised prescription from a medical doctor.

The Russian health‐care system is characterised by the combination of public and private financing along with the provision of compulsory medical insurance (CMI). Within the CMI, some population groups are entitled to the coverage of prescription medicines. However, these groups are limited to patients with rare diseases and low‐income patients. The general population must pay for their own medicines, including antibiotics, out of pocket. Regardless of whether or not patients are entitled to CMI which covers medications, they still must buy a whole package and not only the amount prescribed by a doctor. This situation is the same for public and privet pharmacies.

Russian health‐care system has often been characterised by the multiplicity of informal relations between patients and health‐care workers (Temkina and Rivkin‐Fish 2019; Zvonareva *et al*. 2018). These relationships can be both financial and based on friendship and favours. After the collapse of the Soviet Union, the Russian health‐care systems were severely underfinanced, which stimulated informal payments to doctors and nurses as an important symbol for establishing trusting and caring relationships (Cook 2014, Rivkin‐Fish 2005). By the late 2000 s, informal payments in Russian health care dropped significantly, which was associated, according to Shishkin *et al*. (2014), with the launch of the national priority project Health, implemented in 2006. Health included a salary increase for medical professionals as well as legalisation of various pay‐for‐service practices which would previously have been conducted under the table. Although informal payment practices decreased, Temkina and Rivkin‐Fish (2019) argue that contemporary Russian health care preserves part of its informal organisation. Despite the liberalisation of the health‐care provision that allows patients to choose free or paid medical services, patients continue to rely on personal networks for establishing trusting doctor–patient relationships.

## METHODOLOGY

To understand the work of obligatory medical prescription of antibiotics in health‐care practices, we conducted a qualitative study of the daily antibiotic realities of doctors, pharmacists and patients. To that purpose, we engaged five types of doctors from different specialities: general practitioners (GPs), paediatricians, ENTs, gynaecologists and urologists. Being a part of primary care settings, GPs and paediatricians are in constant contact with communities, thus are primarily responsible for providing information about antibiotic treatments and AMR to patients. In addition, they play a vital role as gatekeepers who refer patients to specialised doctors where more intensive treatment is necessary. At the same time, ENT, gynaecology and urology are medical specialities where the most antibiotics are prescribed. All doctors involved in this study were working in state hospitals and primary care institutions.

Pharmacists were also included as it is common practice in Russia to go to a pharmacist for medical consultations instead of going to a doctor in certain circumstances. Thus, pharmacists also play an important role in informing people about different drugs, their benefits and their potential burdens (Kaae *et al*. 2020, Strachunsky and Andreeva 2004). All pharmacists except one who took part in the study were employed by private organisations as they account for majority of the pharmacological market in Russia.

Patients were engaged in the study to understand how the requirement for a standardised medical prescription of antibiotics had or had not influenced their treatment practices and relationships with doctors and pharmacists. All participating patients were in the process of treatment with one of the medical specialists included in the study. The study took place in a Russian city of medium size between June and August of 2018.

Semi‐structured interviews were conducted to understand antibiotic practices. In total, we conducted 53 interviews: 21 interviews with the doctors—five GPs, four paediatricians, four ENTs, four gynaecologists and four urologists. The age of the participating doctors varied from 25 to 60, and four were male, reflecting the gender distribution of doctors in Russia. The average number of years of experience for the doctors was 19.5. We conducted interviews with 16 pharmacists, aged between 21 and 47; one was male. The average number of years of experience for pharmacists was 14. We conducted 16 interviews with patients, and their age varied from 21 to 78; three were male. Seven were mothers of paediatric patients. The number of interviews conducted was determined by data saturation. All interviews lasted between 15 and 60 minutes.

To recruit doctors and pharmacists for interviews, letters were sent with information about the study to several primary care settings and pharmacies in one Russian city. To the doctors and pharmacists who responded, we provided further details about the aim and methods of the research and obtained informed consent from participants who agreed to give interviews. Patients were recruited from the waiting rooms of the polyclinics. They were provided with detailed information about the study (aim, methods, researchers, their contacts and affiliations). Those patients who agreed to take part in the study signed consent forms. In the case of paediatric patients, parents were invited to take part in the research.

All interviews were conducted in a private space and were subsequently anonymised. Questions in the interviews were developed by an interdisciplinary team of sociologists and medical doctors, with different questions developed for the three groups of participants. Interview guides were tested with the representatives from the three research groups. Based on pilot interviews, the guides were modified for clarity of questions and coherence of the interview. All interviews were voice‐recorded and transcribed verbatim. The language of the interviews was in Russian; quotes in the article were translated into English by the first author.

The study was approved by the ethical committee of the Siberian State Medical University in Russia (№5916 on 22 May 2018).

### Analysis

The data were analysed using thematic analysis (Green and Thorogood 2018). To identify themes and to develop a coding frame, we combined deductive and inductive approaches to analysis. First, some of the key themes were identified at the stage of research design and were used for structuring the interview guides. Those themes were focused on antibiotic prescription practices among medical doctors, antibiotic sale practices among pharmacists and antibiotic use practices among patients. Another key theme that was identified at the stage of research design was participants' attitudes towards the change in the legislation that prohibited the over‐the‐counter sale of antibiotics.

Carefully reading and discussing the interview transcripts in our research team, we conducted an inductive analysis to supplement the previously identified themes. This allowed us to identify themes that describe challenges that different participates indicated as most pressing in the context of changing antibiotic prescription legislation. In addition, we could identify several overarching themes where participates described their informal practices of managing the changing antibiotic policy regulations.

Based on the identified themes, we developed a coding frame that was applied to each interview transcript using the qualitative data software NVivo 9 (QSR International Pty Ltd, Doncaster, Victoria, Australia). The following results section is a report of this thematic analysis.

## RESULTS

### “We cannot always provide quick care”

With the new antibiotic policy of 2017, medical doctors became an obligatory passage point for accessing antibiotics. Referring to the work of Callon (1986) and presenting doctors as an obligatory passage point means that doctors became a point of mediation between patients, pharmacists and antibiotics. The access to antibiotics could not be granted without passing through a doctor's office. The requirement for a standardised medical prescription was seen as re‐articulation of the clinical authority of medical doctors over pharmacists, who were then restricted in providing antibiotics by prescription only. The requirement for a medical prescription can be seen as an attempt to regulate the professional distribution of responsibilities to those who prescribe and those who sell medications. Some of the doctors reflected on this work of medical prescription:

L101: It is very good [that antibiotics can be only sold with prescription]! And not only antibiotics but other medications should also be sold only with prescriptions. Doctors know the value, and patients know the value that without doctors they are nobody. And pharmacists think that they are doctors already. All medication should be sold only with doctors' prescriptions. (ENT, age 34)

P106: Actually, I think that most of the medications should be sold with medical prescriptions because now we have a total absence of authority [*vsedosvolennost*]. And such absence of authority leads to uncontrolled use of medications. (Gynaecologist, age 60)

By giving doctors the ultimate authority over diagnostic and treatment procedures, the prescription became a symbol of evidence‐based decision‐making regarding antibiotic treatment. However, the practices of making this decision have not been changed with the introduction of new policy. Similar to other studies in the field of antibiotic prescription practices, like the research of Broom *et al*. (2014) or Lambert *et al*. (2019), the doctors in our study explained that they based their diagnoses on a combination of experience, personal clinical knowledge and institutionalised clinical recommendations. Apart from this, one of the ENTs explained that as patients would only come to a specialist following a GP's referral, this would be taken as enough evidence that the patient was eligible for antibiotics, and not further laboratory tests would be needed:

L101: You know, patients will not immediately come to an ENT; they will always come from a GP or a paediatrician. They will get treatment at home for a common cold, and then they will come to us with complications. For example, there is no point in treating angina without antibiotics. The same is for sinusitis, it is impossible to treat without antibiotics. (ENT, age 34)

Although the requirement for antibiotic prescription reiterated the authority of doctors over pharmacists, it did not bring any significant systemic changes to their treatment routines. However, the amount of work for doctors actually increased. The requirement for a standardised prescription creates additional paperwork, despite the time for a patient visit remaining unchanged (about 15 min per patient):

U106: Now, apart from medical recommendations that we print on computers, we have to write a prescription. But, we put our stamps and signatures on both of the documents. It takes more time. (GP, age 37)

While no systemic changes occurred in the organisation of health‐care delivery, the standardised medical prescription stimulated an increase in the number of patients seeking medical consultations. Acknowledging this discrepancy in policy attempts to control antibiotics, doctors had to adapt their diagnostic and prescription practices to the increase in the number of patients. This means that they had to navigate the existing limitations of the health‐care system, including the limited amount of medical staff and the limited availability of laboratory testing. Consequently, doctors had to accommodate for both the policy requirements of antibiotic prescription and the realities of health‐care system. As it was shown in our interviews, this resulted in various adaptive and informal practices of antibiotic care:

U107: It happens sometimes that a child got ill on Friday. Saturday and Sunday are holidays, and the child has a high fever. I know that we should wait for 2–3 days [before prescribing antibiotics], but I don't know whether they will call an ambulance or not on the weekend, so I can give them an antibiotic prescription and instruct how to use it in case the child feels worse. Either this way or if they don't use the prescription now, they might use it next time if the child gets very ill. (Paediatrician, age 53)

P205: Well, for example, if we have a patient with acute cystitis. She is in an acute condition at the moment. We will probably not wait for any culturing, so that our poor patient has to wait for this culturing for a week, and even to the point of urinating with blood. Therefore, we have to prescribe a wide range of antibiotics in order to block all kinds of infections and pathogens. (Urologist, age 40)

The introduction of the obligatory medical prescription of antibiotics established medical doctors as an obligatory point of passage to access these medicines and to restrict practices of self‐treatment. It has also increased the workload and pressure on doctors as other elements of the health‐care system have remained unchanged: doctors work to the same consultation time, with no increase in number of medical personnel. In our research, we could see that, instead of restricting self‐treatment with antibiotics, doctors might use their authority to prescribe medicines as a compensatory mechanism against limits of the health‐care system. The requirement for a medical prescription created informal networks of antibiotic care delivery where evidence for an antibiotic prescription included the realities of the current system with its limitations. Therefore, performing a role of an obligatory passage point doctors navigate antibiotic care bridging formal requirements with informal practices.

### “This system had started from the wrong end”

The requirement for a standardised medical prescription restrained the authority of pharmacists to recommend and sell antibiotics. From a policy perspective, this requirement could be seen as a necessary step to reinforce the authority of doctors, whereby they prescribe antibiotics for pharmacists to sell. In practice, however, the obligatory prescription of antibiotics has allowed pharmacists to “observe” the practices and conditions of antibiotic prescription in a hospital. One pharmacist explained that prescriptions were often unclear or lacking particular details and information, leading the pharmacist to resort to guessing the intended medication prescribed by the doctor. Yet, at the same time, the pharmacist was still legally responsible for selling incorrect medicine. One of the pharmacists explained:

F106: Very often doctors confuse forms [of medications]. For instance, cefixime can be in two different forms with different dosages, but they [doctors] only write “cefixime for seven days”. And you have to think and guess – should I give a regular one in capsules where there are six capsules in a box, or another one, where there are seven capsules. In the end, you sell regular capsules because they are cheaper, and people prefer to buy them. But actually, the doctor meant another form. This will result in a return of medication, and this is a conflict because nobody is praising us for returns [returns may cause financial conflicts between pharmacists and the administration of a pharmacy]. And actually, according to the law, we are not allowed to do returns. But it is 600 or 700 rubbles [around 10 euros], and it is essential for people. (Pharmacist, age 28)

In addition, some pharmacists explained that doctors may deviate from standardised prescriptions, yet pharmacists are only allowed to sell standardised prescriptions:

F104: I think that the situation with the requirement for a standardised medical prescription is good, and there should be some connection between a doctor and a pharmacist. But it is important to solve the problem with the standardised prescription forms because patients often comment that doctors don't have those. (Pharmacist, age 32)

These examples indicate an interesting moment in the work of prescriptions. While originally intended to separate the responsibilities of doctors and pharmacists, they instead re‐mediated the relationship, where pharmacists can observe certain pitfalls in the work of doctors, including the lack of necessary material infrastructures like prescription forms. Although medical doctors are established as an official obligatory passage point for access to antibiotics, it is pharmacists who are positioned as mediators between doctors and patients. In the interviews, they reflected on this mediating role through the stories of their clients:

F101: [Q: for what reasons do people ask to sell them antibiotics without a prescription?] They don't have time to go to a doctor, that is the first reason. There is a very long wait time to get an appointment with a doctor. For example, you cannot get to an ENT specialist because you need to have an appointment a month in advance. A child might have an acute exacerbation of sinusitis right now, but an appointment with an ENT specialist is only in a month. Also, it is impossible to go to a private doctor, for instance, because it is expensive. Actually, I think that this system [of control over the sale of antibiotics] started from the wrong end because it is necessary to first fix the system of appointments with doctors so that they [patients] can get a consultation the same day they need it. (Pharmacist, age 35)

The requirement for a standardised medical prescription has affected the communication between pharmacists and their clients. Several pharmacists described that since the introduction of standardised prescriptions and implementation of surprise inspections they have been distinguishing between their formal communication with new patients and informal communication with familiar patients. One pharmacist elaborated that familiar relationships with a customer may blur the line between the role of a doctor and the role of a pharmacist. In this situation, a pharmacist performed both diagnostics and selection of medical therapy:

F103: To tell the truth, if it is our regular customer, maybe a child of a co‐worker, then we can [sell antibiotics without a prescription]. So if there are no unfamiliar people in a line, if I am alone with a client, then I can do it [sell antibiotics without a prescription]. But if I see a person the first time, then of course not. (Pharmacist, age 20)

Therefore, the requirement for a standardised prescription had stimulated the emergence of a grey zone or a hidden network of antibiotic care, where antibiotics are managed between a pharmacist and a patient without the control of the state or involvement of a doctor. This hidden network, however, was not presented by pharmacists as a revolt or resistance to the policy requirement for antibiotic prescription or a medical authority of doctors. Rather, it was justified as a necessary compensatory and even caring response to the current gaps in the infrastructure of health‐care delivery, including the lack of doctors in hospitals which negatively impacted timely delivery of care. It was presented as a form of care for patients whose social and economic situation might not allow them to take time off work to go to a doctor:

F102: There are sometimes cases when we [pharmacists] recommend prescribed medications, but it is usually, for example, eye drops or something like that. Something for what a patient would not go to a doctor, but of course all antibiotics for eyes can only be sold with a prescription. But in these cases, we take responsibility because a person with conjunctivitis [eye infection] will not go to a doctor and will infect other people. (Pharmacist, age 30)

The introduction of standardised medical prescriptions for antibiotics in the practice of pharmacists has made visible the tensions between the work of doctors and the needs of patients. Although unable to officially recommend and sell antibiotics, pharmacists can observe the infrastructural realities of doctors and highlight the gaps in these infrastructures through the use of standardised medical prescriptions. The standardised medical prescriptions coordinate the official channels of communication between a pharmacist and a patient; however, similar to the situation with medical doctors, they stimulate the creation of informal networks of antibiotic care. These informal networks can be understood as a response to AMR control programmes that do consider the limits of health‐care infrastructures.

### “Not everyone can take a day off work”

The presentation of patients as those who exercise undesirable behaviours of self‐treatment through the use of antibiotics is common in policy discourses on AMR (Haenssgen *et al*. 2018, Will 2018). In this context, the requirement for a medical prescription functions as a technique to prevent this behaviour. However, as studies of Rodrigues (2020) and Willis and Chandler (2019) show, self‐treatment with antibiotics is not a question of choice but is influenced by social, economic and political processes, such as access and affordability of health care, non‐therapeutic forms of care, and the opportunity to take time from work to visit a doctor and convalesce. In our interviews with patients in Russia, we observed similar processes.

Some of the patients saw the requirement for a prescription as a positive move that would protect people from the overuse of antibiotics. For instance, one of the patients told us that “the body of each person is different, and it means that you must do it [take antibiotics] only under a doctor's supervision” (P110, age 42). Other participants explained that it was difficult for them to accept the new requirements. Complexities regarding long waiting times for clinical appointments, mentioned by both doctors and pharmacists, were further elaborated upon by patients. Having an appointment with a doctor would mean long waiting times and the necessity of taking a day off work, which was a significant obstacle for some patients:

PT101: To tell the truth, when it all just started [stricter requirements for antibiotic prescriptions], I did not like it. I did not like that it was now necessary to go to a doctor every time. (Patient, age 31)

PT102: Now I am ok about it [stricter requirements for antibiotic prescriptions], but when this requirement first took place [I did not like it]. Before you could just go and buy medications, because for some it was difficult to go for an appointment with a doctor; not everyone can take a day off work. (Patient, age 30)

One patient was particularly critical about antibiotic prescription requirements. She explained that instead of going to a doctor she preferred to rely on her pharmacist. She explained that a visit to pharmacy would save her time as well we money. She argued that medical doctors would only prescribe her expensive medications, this despite the fact that doctors in Russia are obligated to prescribe medicines based on their active substances rather than the brand names:

PT107: It [amoxicillin] was once prescribed to me by a doctor. But it was a long time ago. Then I started to always buy it in a pharmacy; it is not expensive. And now it is very bad that I can buy it only with a prescription. It is really terrible for me! Because now you need to go to a doctor, pay money, and a doctor will prescribe you very expensive medications, very expensive – he will not prescribe a cheap one! And then I would have to run to a pharmacy. (Patient, age 49)

PT107: If I feel that I am getting ill and I have an important deadline, you know, I am an accountant, then I can of course increase the dosage (of amoxicillin) for myself as a prophylactic measure. (Patient, age 49)

While the requirement for a medical prescription aims to regulate the biological body of a patient and prevent self‐treatment behaviour, the economic body of the patient perceives antibiotics as a requirement to stay productive in society, to earn money and to prevent poverty. In our interviews with Russian patients, we identified that similar to the work by Pearson and Chandler (2019), antibiotics in Russia can be understood as an economic tool, or a “quick fix” that can fix the body to make it productive again. As it can be seen from the quote above, some patients believe that they cannot allow themselves to be ill if they have important deadlines at work.

Adapting to the new conditions which require a visit to a doctor as an obligatory passage point to access antibiotics, some patients explained that they would prefer to use leftover antibiotics rather than go to a doctor if their health conditions were not severe. Most of the patients from our study had leftover antibiotics at home from their previous prescriptions. Patients incorporated themselves into the informal networks of care with doctors and pharmacists as an additional safeguard that they can receive antibiotic treatment regardless of the health‐care system restrains and limits. A mother of a paediatric patient described a situation that was similar to the one described by one of the doctors—if a patient got ill on the weekend, she would not be able to receive specialised care on Saturday or Sunday. In this scenario, patients preferred to not wait until they would be able to see a doctor, but to start treatment with antibiotics if they had a chance:

PT101: For example, it was a case that we had some leftover antibiotics. He [a child] recently had a tooth problem – he had gumboil. And it was a weekend and nobody could receive us [at a hospital]. It was 4 pm, and they receive patients only until 2 pm. I have some friends who are clinicians and they recommended me to give him antibiotics to prevent any complications. And I had those leftover antibiotics, and I gave him one pill. Then, on Sunday, we came in an ambulance to a paediatric dental clinic. They told us there that we did good to give him an antibiotic. (Patient, age 31)

These informal practices and networks of antibiotic care can be conceptualised as compensatory and adaptive mechanisms to the limited functionality and rigidity of antibiotic control policies. They are not necessarily a form of resistance or a request for an open antibiotic market, but rather they are symptoms of poorly functioning official channels of antibiotic management that do not reflect the realities of health‐care system and its patients. This is supported by a fact that most of our participants were very cautious about antibiotics and their effects. Contrary to some studies arguing that patients demand antibiotics from doctors (Ashworth *et al*. 2016, Little *et al*. 2004), participants in our study expressed that they would prefer to avoid antibiotic treatment where possible as they considered them to be strong medicine with potentially serious side effects. Those patients explicitly asked doctors to not prescribe them antibiotics and requested alternative treatment:

PT102: If it is for kids, I ask whether it is possible to have treatment without antibiotics. Because I am worried. They probably have some side effects, such as milk fever or something like that. (Patient, age 30)

PT103: [Q: Have you ever disputed a doctor's prescriptions?] Yes, I asked for something else. I do not feel so bad as to need antibiotics. (Patient, age 67)

Aiming to control the practices of self‐treatment, the requirement for a standardised medical prescription depicts patients as potentially irresponsible users. However, our research shows that patients' relationships with antibiotics reflect the broader social and economic realities of their life, rather than a simple lack of knowledge. Antibiotics were perceived as a necessity for maintaining functionality in society, while at the same time many patients were cautious about using them. While the introduction of the prescription required patients to coordinate their treatment practices with a doctor and a pharmacist, existing limits in health‐care delivery could not accommodate this requirement, leading to the creation of informal channels of coordination between these actors.

## INFORMAL INFRASTRUCTURE TO COMPENSATE FOR POLICY GAPS

In this article, we studied how the requirement for a medical prescription had affected practices of doctors, pharmacists and patients. The analysis showed that while this requirement aimed to establish a clear distribution of responsibilities between doctors and pharmacists to prevent patients' self‐treatment, in practice it unpacked the infrastructural gaps that stimulate the development of informal antibiotic care networks. The requirement for medical prescriptions obliged patients to seek an appointment with doctors instead of going to the pharmacy to buy antibiotics. However, our analysis reveals that this increased the pressure on the limited number of medical staff and stimulated the practice of just‐in‐case prescriptions of antibiotics by doctors. In tandem, the system of over‐the‐counter sales of antibiotics did not disappear but instead became more clandestine, accessible only to friends and acquaintances of pharmacists. These informal practices can be conceptualised as compensating for infrastructural gaps that could not be resolved by a requirement for antibiotic prescription, such as the limited number of doctors and corresponding insufficient availability of medical appointments, lack of official forms for prescriptions, and the constrained economic situation of patients that does not allow them to take time off work to visit a doctor.

The insights from our study confirmed the arguments of other scholars who described an infrastructural embeddedness of antibiotic practices. Similar to the study of Broom *et al*. (2017), we could see that doctors, as well as pharmacists, adapted their antibiotic practices to the infrastructural conditions that might hinder patients' access to timely medical appointments. Also, along with the study by Willis and Chandler (2019), some of our patients understood antibiotics as quick fixes that enabled faster recovery and return to their social and economic responsibilities. However, our study demonstrated that antibiotic practices are not driven by economic factors exclusively. Rather, the organisation of health‐care delivery (e.g. the timetable for when the ambulance is working) and structural limits for access to certain specialists stimulated medical doctors to prescribe precautionary antibiotics in case a patient would not be able to access timely care. In addition, several of our patients–participants expressed that they would prefer a non‐antibiotic treatment if they had a choice as they were worried about the potential side effects of these medicines. While the study of Little *et al*. (2004) demonstrated that doctors perceive pressure from patients to prescribe antibiotics; our findings showed that patients themselves would prefer alternative ways of treatment.

Analysing how AMR is constructed as a problem in public health, Chandler (2019) stresses that antibiotic practices are shaped by the infrastructural conditions in which they are situated, including material organisation of care delivery as well as adaptive relations between different health actors. Understanding practices of doctors, pharmacists and patients through the lens of health‐care infrastructure provides insights into the obligatory medical prescription of antibiotics as a boundary object of such infrastructure, coordinating and re‐aligning the work of the main actors.

In the context of new antibiotic policies to reduce AMR in Russia, the requirement for a standardised medical prescription can be seen as a boundary object facilitating the coordination between doctors, pharmacists and patients. However, some of this coordination work takes place outside of the officially established infrastructures to informally compensate for the gaps in care delivery. Imposed to coordinate new hierarchical relationships between patients, doctors and pharmacists, the obligatory medical prescription unpacks the current political economy in Russia. In this system, doctors and pharmacists informally accommodate for the limits in health‐care system and for the socioeconomic conditions of patients who cannot afford to take time off work to be sick. The actual coordination of obligatory antibiotic prescriptions escapes the hierarchy between medical doctors, pharmacists and patients when it comes to diagnosis and treatment. By navigating the antibiotic prescriptions, doctors, pharmacists and patients informally compensate for the gaps in the existing infrastructure. Following these informal practices, we could map the inconsistencies in the current policy approach to tackle AMR as a behavioural rather than infrastructural problem.

While international and national policies continue to initiate behavioural interventions aimed at antibiotic users and prescribers, users have to adopt practices to fix the gaps that become visible through such interventions. The frictions between the policy understanding of AMR and its everyday practical articulations indicate a gap between policy and daily realities of health‐care actors. While the former imagines AMR as problem defined by the lack of knowledge among antibiotic users, the latter defines it as a consequence of economic and health‐care infrastructures that limits a patients' ability to be sick and take time off work.

In his study of pasteurisation in France, Latour (1988) argues that for an intervention to work, it has to be aligned with everything that allows it to work, in a process of mutual translation—the co‐production of ideas and practices. In other words, to make AMR policies more efficient, the conditions that allow them to be efficient have to be adapted through the cooperation of local and national actors. As we saw in our research, the informal practices involving doctors, pharmacists and patients indicated the need for infrastructural changes such as the increased availability of medical staff, increased time for a medical appointment and legal protections for patients as economic subjects that could fall ill and needed time for recovery. To reassemble the socioeconomic and temporal infrastructures that influence antibiotic practices, it is important to take seriously the realities and practicalities that are communicated by public health professionals and patients.

## AUTHOR CONTRIBUTION

**Alena Kamenshchikova:** Conceptualization (lead); data curation (equal); formal analysis (lead); writing – original draft (lead); writing – review and editing (lead). **Marina M. Fedotova:** Data curation (equal); resources (equal); writing – review and editing (equal). **Olga S. Fedorova:** Data curation (equal); resources (equal). **Sergey V. Fedosenko:** Data curation (equal); resources (equal). **Petra F.G. Wolffs:** Supervision (equal); writing – review and editing (equal). **Christian J.P.A. Hoebe:** Supervision (equal); writing – review and editing (equal). **Klasien Horstman:** Conceptualization (equal); supervision (equal); writing – review and editing (equal).
